# Survival outcomes and clinical benefit in patients with acute myeloid leukemia treated with glasdegib and low-dose cytarabine according to response to therapy

**DOI:** 10.1186/s13045-020-00929-8

**Published:** 2020-07-14

**Authors:** Jorge E. Cortes, Florian H. Heidel, Walter Fiedler, B. Douglas Smith, Tadeusz Robak, Pau Montesinos, Anna Candoni, Brian Leber, Mikkael A. Sekeres, Daniel A. Pollyea, Roxanne Ferdinand, Weidong Wendy Ma, Thomas O’Brien, Ashleigh O’Connell, Geoffrey Chan, Michael Heuser

**Affiliations:** 1 Georgia Cancer Center, Augusta, CA USA; 2 Otto-von-Guericke University Medical Center Magdeburg, Magdeburg, Germany; 3 Internal Medicine II, University Hospital Jena, Jena, Germany; 4 Department of Hematology and Oncology, University Hospital Hamburg-Eppendorf, Hamburg, Germany; 5 Johns Hopkins Sidney Kimmel Comprehensive Cancer Center, Baltimore, MD USA; 6 Department of Hematology, Medical University of Łódź and Copernicus Memorial Hospital, Łódź, Poland; 7 Hospital Universitari i Politècnic La Fe, Valencia, Spain; CIBERONC, Instituto Carlos III, Madrid, Spain; 8 Azienda Sanitaria Universitaria Integrata di Udine, Udine, Italy; 9 Juravinski Hospital at Hamilton Health Sciences, Hamilton, ON Canada; 10 Leukemia Program, Cleveland Clinic, Cleveland, OH USA; 11 Division of Hematology, University of Colorado School of Medicine, Aurora, CO USA; 12 Pfizer Oncology, New York, NY USA; 13 Department of Hematology, Hemostasis, Oncology and Stem Cell Transplantation, Hannover Medical School, Hannover, Germany

**Keywords:** Glasdegib, Acute myeloid leukemia, Clinical trial, Disease response, Efficacy

## Abstract

**Background:**

The phase 2 BRIGHT AML 1003 trial evaluated efficacy and safety of glasdegib + low-dose cytarabine (LDAC) in patients with acute myeloid leukemia ineligible for intensive chemotherapy. The multicenter, open-label study randomized patients to receive glasdegib + LDAC (*n* = 78) or LDAC alone (*n* = 38). The rate of complete remission (CR) was 19.2% in the glasdegib + LDAC arm versus 2.6% in the LDAC arm (*P* = 0.015).

**Methods:**

This post hoc analysis determines whether the clinical benefits of glasdegib are restricted to patients who achieve CR, or if they extend to those who do not achieve CR.

**Results:**

In patients who did not achieve CR, the addition of glasdegib to LDAC improved overall survival (OS) versus LDAC alone (hazard ratio = 0.63 [95% confidence interval, 0.41–0.98]; *P* = 0.0182; median OS, 5.0 vs 4.1 months). Additionally, more patients receiving glasdegib + LDAC achieved durable recovery of absolute neutrophil count (≥ 1000/μl, 45.6% vs 35.5%), hemoglobin (≥ 9 g/dl, 54.4% vs 38.7%), and platelets (≥ 100,000/μl, 29.8% vs 9.7%). Transfusion independence was achieved by 15.0% and 2.9% of patients receiving glasdegib + LDAC and LDAC alone, respectively.

**Conclusions:**

Collectively, these data suggest that there are clinical benefits with glasdegib in the absence of CR.

**Trial registration:**

ClinicalTrials.gov NCT01546038 (March 7, 2012)

## Background

Glasdegib, an oral, small-molecule inhibitor of the Smoothened (SMO) receptor, selectively inhibits the Hedgehog signaling pathway. In a phase 2 randomized study that included patients with newly diagnosed acute myeloid leukemia (AML) who were ineligible for intensive chemotherapy (BRIGHT AML 1003), the addition of glasdegib to low-dose cytarabine (LDAC) demonstrated superior overall survival (OS) compared with LDAC alone (hazard ratio [HR] = 0.51; 80% confidence interval [CI], 0.39–0.67; *P* = 0.0004; median OS 8.8 vs 4.9 months). The combination of glasdegib and LDAC was generally well tolerated, with a manageable safety profile, consistent with findings in older patients receiving chemotherapy, and with “class effect” toxicities reported for other marketed SMO inhibitors. The combination did not appear to increase cytopenias, bleeding, or infection [1]. Based on the findings from BRIGHT AML 1003, glasdegib was approved in the USA, in combination with LDAC, for the treatment of newly diagnosed AML in patients unable to receive intensive chemotherapy due to comorbidities or age (≥ 75 years) [2].

Despite a significantly higher response rate with glasdegib + LDAC compared with LDAC alone, the majority of patients in the BRIGHT AML 1003 study did not achieve complete remission (CR) [1]. Given the importance of the Hedgehog signaling pathway in the maintenance of leukemic stem cells (LSCs), but not normal adult hematopoiesis, and the effect of glasdegib on LSCs, we hypothesized that there would be clinical benefits for patients treated with glasdegib regardless of whether they achieved a CR or not [3–6]. We thus performed this post hoc analysis to determine whether the survival and clinical benefits of glasdegib are restricted to patients who achieve CR or if it extends to those who do not achieve CR.

## Methods

### Study design and patients

BRIGHT AML 1003 (ClinicalTrials.gov, NCT01546038) was an open-label, randomized, multicenter, phase 2 study for which the methods have previously been published [1]. Briefly, BRIGHT AML 1003 enrolled patients aged ≥ 55 years with newly diagnosed, previously untreated AML or high-risk myelodysplastic syndromes (World Health Organization 2008 classification) who were ineligible for intensive chemotherapy. Ineligibility for intensive chemotherapy was defined as meeting one or more of the following criteria: age ≥ 75 years, serum creatinine > 1.3 mg/dl, severe cardiac disease (e.g., left ventricular ejection fraction < 45% by multigated acquisition or echocardiography at screening), or Eastern Cooperative Oncology Group performance status = 2. Patients were randomized 2:1 to receive glasdegib + LDAC or LDAC alone. Glasdegib 100 mg once daily was administered orally in 28-day cycles on a continuous basis, and LDAC 20 mg was administered subcutaneously twice daily for 10 days, every 28 days, until disease progression, unacceptable toxicity, or patient refusal. This analysis assessed efficacy and safety in patients with AML only; the data cut-off date was October 2018.

The study was conducted in accordance with the Declaration of Helsinki. All patients provided written informed consent before study procedures began, and the protocol was approved by institutional review boards at each study site.

### Efficacy and safety assessments

This post hoc exploratory analysis assessed efficacy and safety in patients with AML who achieved CR and those who did not achieve CR (including patients with CR with incomplete hematologic response [CRi]) at any point during treatment. Response to treatment was assessed based on the International Working Group response criteria for AML [7]. CR was defined as < 5% bone marrow blasts, no extramedullary disease, absolute neutrophil count (ANC) ≥ 1000/μl, platelet count ≥ 100,000/μl, and transfusion independent. CRi was defined as < 5% bone marrow blasts, no extramedullary disease, and with either neutrophils or platelets not recovered to CR levels. Bone marrow samples for response assessment were collected at screening; on cycle 3, day 1; and every third cycle, within 14 days of achieving initial hematologic recovery in the peripheral blood (defined as ANC > 1000/μl and platelets ≥ 100,000/μl), at end of treatment, and at the investigator’s discretion (± 7 days of nominal time).

Safety assessments included adverse events (AEs), classified and graded based on the National Cancer Institute Common Terminology Criteria for Adverse Events v4.0, based on history and physical exam, vital signs, laboratory evaluations, and 12-lead electrocardiograms.

### Statistical analysis

After discontinuation of study treatment, patients were followed until death or for 4 years from first dose. OS was defined as the time from date of randomization to death from any cause. Patients not known to have died at the last follow-up were censored on the date they were last known to be alive. OS was estimated using the Kaplan–Meier method. Transfusion independence was defined as ≥ 8 consecutive weeks without red blood cell and/or platelet transfusion. Safety data were summarized descriptively and included all randomized patients who received at least one dose of any of the study medications.

## Results

### Patients and duration of treatment

A total of 116 patients with AML were randomized to receive glasdegib + LDAC (*n* = 78) or LDAC alone (*n* = 38); among them, 75 and 36 patients received study treatments, respectively (Additional file 5: Table S1). Response rates for all patients are shown in Table 1. The response rate was significantly higher for patients who received glasdegib + LDAC compared with LDAC alone; the respective rate of CR was 19.2% versus 2.6%; *P* = 0.015. Patient demographics and baseline characteristics are summarized in Table 2. Of the patients who achieved CR and those who did not achieve CR, the median age at study entry was ≥ 74 years in the glasdegib + LDAC and LDAC alone arms, and more than half of patients in each subgroup were male.

**Table 1:** Best overall response for patients with AML at any time on treatment

Best overall response, *n* (%)	Glasdegib + LDAC *N* = 78	LDAC alone *N* = 38
**Achieved CR**		
CR	15 (19.2)	1 (2.6)
**Did not achieve CR**		
CRi	4 (5.1)	1 (2.6)
PR	5 (6.4)	0
PRi	2 (2.6)	0
MLFS	2 (2.6)	0
MR	4 (5.1)	4 (10.5)
SD	14 (17.9)	9 (23.7)
Treatment failure	9 (11.5)	7 (18.4)
Not evaluable	23 (29.5)	16 (42.1)

*AML* acute myeloid leukemia, *CR* complete remission, *CRi* CR with incomplete hematologic recovery, *LDAC* low-dose cytarabine, *MLFS* morphologic leukemia-free state, *MR* minor response, *PR* partial remission, *PRi* PR with incomplete hematologic recovery, *SD* stable disease

**Table 2:** Patient demographics and baseline characteristics

	Achieved CR		Did not achieve CR	
Characteristic	Glasdegib + LDAC *N* = 15	LDAC alone *N* = 1	Glasdegib + LDAC *N* = 63	LDAC alone *N* = 37
Age (years), *n* (%)				
45–64	0	0	1 (1.6)	1 (2.7)
≥ 65	15 (100)	1 (100)	62 (98.4)	36 (97.3)
Median (range)	74 (65–87)	78 (78–78)	77 (64–92)	76 (58–83)
Sex, *n* (%)				
Female	5 (33.3)	1 (100)	14 (22.2)	14 (37.8)
Male	10 (66.7)	0	49 (77.8)	23 (62.2)
ECOG PS, *n* (%)				
0	0	1 (100)	10 (15.9)	2 (5.4)
1	5 (33.3)	0	21 (33.3)	17 (45.9)
2	10 (66.7)	0	31 (49.2)	18 (48.6)
Not reported	0	0	1 (1.6)	0
Cytogenetic risk, *n* (%)				
Good/intermediate risk	12 (80.0)	0	41 (65.1)	22 (59.5)
Poor risk	3 (20.0)	1 (100)	22 (34.9)	15 (40.5)
ELN risk stratification, *n* (%)				
Favorable	1 (6.7)	0	4 (6.3)	3 (8.1)
Intermediate I	8 (53.3)	0	19 (30.2)	11 (29.7)
Intermediate II	3 (20.0)	0	18 (28.6)	8 (21.6)
Adverse	3 (20.0)	1 (100)	22 (34.9)	15 (40.5)
Disease history, *n* (%)				
De novo	7 (46.7)	1 (100)	31 (49.2)	17 (45.9)
Secondary AML	8 (53.3)	0	32 (50.8)	20 (54.1)
Mutations, *n* (%)*				
*FLT3*^†^	0	0	5 (7.9)	0
*IDH1* or *IDH2*	4 (26.7)	0	15 (23.8)	6 (16.2)
*NPM1*	1 (6.7)	0	4 (6.3)	1 (2.7)
Unknown	4 (26.7)	0	16 (25.4)	13 (35.1)

*AML* acute myeloid leukemia, *CR* complete remission, *ECOG PS* Eastern Cooperative Oncology Group performance status, *ELN* European LeukemiaNet, *LDAC* low-dose cytarabine

*Baseline gene mutations were determined in 58/78 patients receiving glasdegib + LDAC (CR, *n* = 11; no CR, *n* = 47) and 25/38 patients receiving LDAC alone (CR, *n* = 1; no CR, *n* = 24)

^†^Includes only *FLT3* point mutations

Among patients who achieved CR, the median duration of treatment was 17 (range, 1–44) cycles with glasdegib + LDAC and seven cycles for the one patient receiving LDAC alone. Of the patients who did not achieve CR, the median duration of treatment was two (range, 1–43) cycles with glasdegib + LDAC and two (range, 1–9) cycles with LDAC alone; 48.3% and 37.1% of patients received ≥ 3 cycles of treatment, respectively, while 28.3% and 8.6% of patients received ≥ 6 cycles. The duration of treatment with best overall response is shown for individual patients in the Additional file 1: Fig. S1. Following ≥ 6 cycles of treatment, two patients achieved CR (cycles 6 and 32) and three patients achieved CRi (cycle 6) in the glasdegib + LDAC arm; one patient achieved CRi (cycle 6) in the LDAC alone arm. The most common reason for treatment discontinuation among all patient cohorts was insufficient clinical response.

Median follow-up for OS for all patients was 43.4 months with glasdegib + LDAC and 42.0 months with LDAC alone.

### Efficacy

#### Patients who achieved CR

The median time to CR was 59 (range, 33–919) days with glasdegib + LDAC and 170 days for the one patient receiving LDAC alone; the median duration of CR was 302 (range, 1–1262) days and 91 days, respectively.

**Fig. 1:**
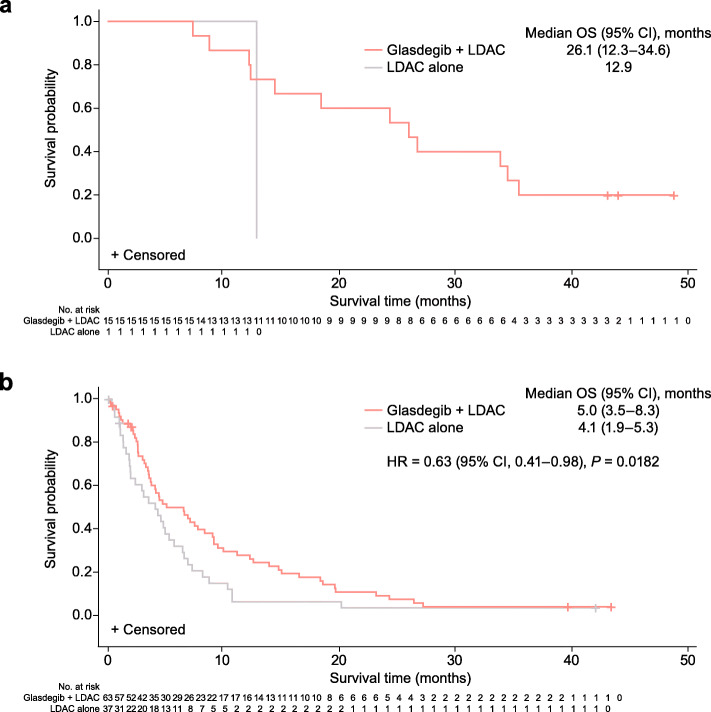
Kaplan–Meier plots of OS. **a** In patients who achieved CR. **b** In patients who did not achieve CR. Abbreviations: CI, confidence interval; CR, complete remission; HR, hazard ratio; LDAC, low-dose cytarabine; OS, overall survival

For patients who achieved CR, the median OS was 26.1 (95% CI, 12.3–34.6) months with glasdegib + LDAC and 12.9 months with LDAC alone (Table 3 and Fig. 1). The 12-month survival probability was 86.7% (95% CI, 56.4–96.5) for glasdegib + LDAC; the one patient with LDAC alone died at 12.9 months. The cause of death was AML in 66.7% of patients receiving glasdegib + LDAC and in the one patient receiving LDAC alone. Among patients who achieved CR, the median OS for patients receiving glasdegib + LDAC was longer in patients with secondary AML than with de novo AML (34.3 months and 14.5 months, respectively (Table 4)).

**Table 3:** OS in patients who achieved CR and those who did not achieve CR

	Achieved CR		Did not achieve CR	
	Glasdegib + LDAC *n* = 15	LDAC alone *n* = 1	Glasdegib + LDAC *n* = 63	LDAC alone *n* = 37
Median OS, months (95% CI)	26.1 (12.3–34.6)	12.9 (N/E–N/E)	5.0 (3.5–8.3)	4.1 (1.9–5.3)
Survival probability, % (95% CI)				
6 months	100 (100–100)	100 (100–100)	49.5 (36.3–61.5)	31.5 (17.2–47.0)
12 months	86.7 (56.4–96.5)	100 (100–100)	27.3 (16.7–39.0)	5.7 (1.0–16.8)
Deaths, *n* (%)				
Cause of death: disease under study	10 (66.7)	1 (100)	49 (77.8)	28 (75.7)

*CI* confidence interval, *CR* complete remission, *LDAC* low-dose cytarabine, *N/E* not evaluable, *OS* overall survival

**Table 4:** OS by de novo and secondary AML

	De novo		Secondary AML	
	Glasdegib + LDAC	LDAC alone	Glasdegib + LDAC	LDAC alone
**Achieved CR**	***n* = 7**	***n* = 1**	***n* = 8**	***n* = 0**
Median OS, months (95% CI)	14.5 (8.8–26.1)	12.9 (N/E–N/E)	34.3 (7.4–N/E)	–
Survival probability, % (95% CI)				
6 months	100 (100–100)	100 (100–100)	100 (100–100)	–
12 months	85.7 (33.4–97.9)	100 (100–100)	87.5 (38.7–98.1)	–
Deaths, *n* (%)	6 (85.7)	1 (100)	6 (75.0)	–
Cause of death: disease under study	5 (71.4)	1 (100)	5 (62.5)	–
**Did not achieve CR**	***n* = 31**	***n* = 17**	***n* = 32**	***n* = 20**
Median OS, months (95% CI)	4.4 (2.6–6.9)	3.9 (1.3–8.7)	7.5 (3.4–9.5)	4.1 (1.5–6.4)
Survival probability, % (95% CI)				
6 months	39.9 (22.1–57.1)	31.3 (11.4–53.6)	58.2 (39.1–73.2)	31.9 (13.1–52.6)
12 months	29.0 (13.8–46.1)	12.5 (2.1–32.8)	25.9 (12.2–41.9)	N/E (N/E–N/E)
Deaths, *n* (%)	27 (87.1)	15 (88.2)	30 (93.8)	19 (95.0)
Cause of death: disease under study	23 (74.2)	11 (64.7)	26 (81.3)	17 (85.0)

*AML* acute myeloid leukemia, *CI* confidence interval, *CR* complete remission, *LDAC* low-dose cytarabine, *N/E* not evaluable, *OS* overall survival

#### Patients who did not achieve CR

Among the patients who did not achieve CR, OS was longer with glasdegib + LDAC (*n* = 63) versus LDAC alone (*n* = 37) with HR = 0.63 (95% CI, 0.41–0.98), *P* = 0.0182; the median OS was 5.0 (95% CI, 3.5–8.3) months and 4.1 (95% CI, 1.9–5.3) months, respectively (Table 3 and Fig. 1). The respective 12-month survival probability was 27.3% (95% CI, 16.7–39.0) and 5.7% (95% CI, 1.0–16.8). Disease progression was the cause of death in 77.8% of patients receiving glasdegib + LDAC and in 75.7% of patients receiving LDAC alone.

To try to account for the possible effect of early mortality versus treatment effect, OS was assessed in patients who did not achieve CR and received ≥ 56 days of therapy. OS was longer with glasdegib + LDAC (*n* = 36) versus LDAC alone (*n* = 13) with HR = 0.54 (95% CI, 0.27–1.05), *P* = 0.033, and median OS 8.3 (95% CI, 4.4–13.9) months and 5.3 (95% CI, 4.1–6.5) months, respectively. The respective 12-month survival probability was 42.9% (95% CI, 26.4–58.3) and 7.7% (95% CI, 0.5–29.2), respectively. Survival rates for patients who did not achieve either CR or CRi are shown in Additional file 2: Fig. S2.

Among patients who did not achieve CR, 27 (45.0%) in the glasdegib + LDAC arm and 10 (28.6%) in the LDAC alone arm were reported to have received follow-up systemic therapies after discontinuation of study treatment. When patients were censored at the start of follow-up systemic therapies, the median OS was 7.1 (95% CI, 3.5–14.7) months with glasdegib + LDAC and 3.5 (95% CI, 1.9–4.9) months with LDAC alone (HR = 0.52 [95% CI, 0.30–0.89]; *P* = 0.008). The respective 12-month survival probability was 39.0% (95% CI, 22.9–54.9) and 7.6% (95% CI, 0.7–26.6) (Additional file 3: Fig. S3).

Analyzing patients who did not achieve CR with regard to either de novo or secondary AML, the median OS was again longer in patients receiving glasdegib + LDAC versus LDAC alone (Table 4). Acknowledging the small numbers in this subset analysis, the improvement in OS with glasdegib + LDAC was also consistent across cytogenetic risk groups (Table 5). The greatest improvement in OS was seen among patients with good/intermediate cytogenetic risk; median OS, 7.7 (95% CI, 3.5–11.1) months with glasdegib + LDAC and 5.3 (95% CI, 3.5–8.7) months with LDAC alone. For patients with poor cytogenetic risk, the median OS was 4.0 (95% CI, 1.9–4.7) months and 1.8 (95% CI, 0.6–3.1) months with glasdegib + LDAC and LDAC alone, respectively.

**Table 5:** OS by cytogenetic risk

	Good/intermediate		Poor	
	Glasdegib + LDAC	LDAC alone	Glasdegib + LDAC	LDAC alone
**Achieved CR**	***n* = 12**	***n* = 0**	***n* = 3**	***n* = 1**
Median OS, months (95% CI)	30.4 (14.5–N/E)	–	8.8 (7.4–12.4)	12.9 (N/E–N/E)
Survival probability, % (95% CI)				
6 months	100 (100–100)	–	100 (100–100)	100 (100–100)
12 months	100 (100–100)	–	33.3 (0.9–77.4)	100 (100–100)
Deaths, *n* (%)				
Cause of death: disease under study	7 (58.3)	–	3 (100)	1 (100)
**Did not achieve CR**	***n* = 41**	***n* = 22**	***n* = 22**	***n* = 15**
Median OS, months (95% CI)	7.7 (3.5–11.1)	5.3 (3.5–8.7)	4.0 (1.9–4.7)	1.8 (0.6–3.1)
Survival probability, % (95% CI)				
6 months	60.7 (43.5–74.1)	45.1 (23.2–64.8)	29.0 (11.9–48.7)	13.3 (2.2–34.6)
12 months	34.3 (19.9–49.2)	10.0 (1.7–27.3)	14.5 (3.6–32.5)	N/E (N/E–N/E)
Deaths, *n* (%)				
Cause of death: disease under study	30 (73.2)	15 (68.2)	19 (86.4)	13 (86.7)

*CI* confidence interval, *CR* complete remission, *LDAC* low-dose cytarabine, *N/E* not evaluable, *OS* overall survival

**Fig. 2:**
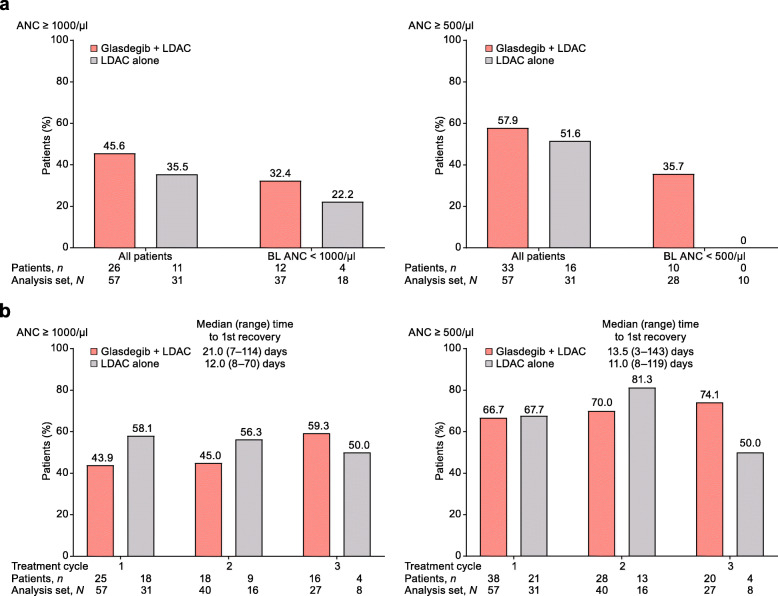
ANC recovery in patients who did not achieve CR. **a** Percentage of patients with durable (≥ 2 consecutive visits) recovery at any time on study. **b** Percentage of patients with ANC recovery after the first, second, and third treatment cycle. For treatment cycle analysis, one threshold measurement was required; all patients were included regardless of their BL levels but each cycle only included remaining patients at risk in that cycle. Analysis set, *N* = number of patients with ANC results in the cycle; patients, *n* = number of patients meeting recovery criteria in the cycle. Abbreviations: ANC, absolute neutrophil count; BL, baseline; CR, complete remission; LDAC, low-dose cytarabine

**Fig. 3:**
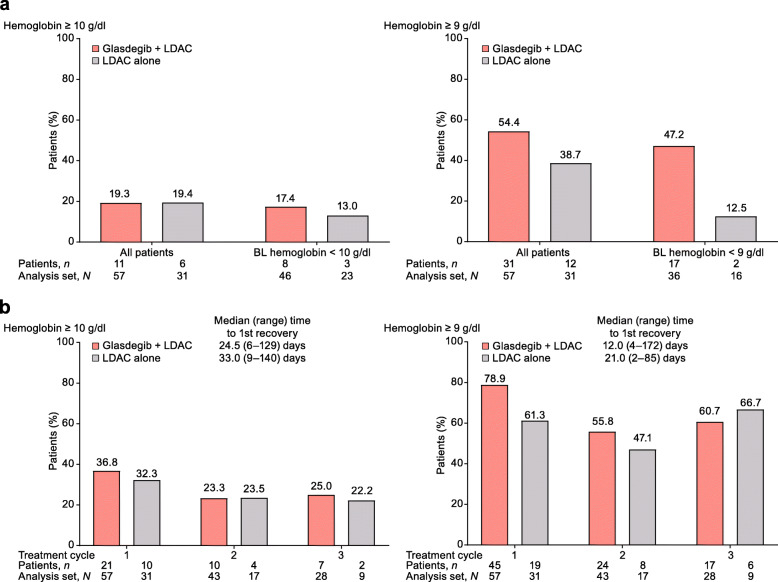
Hemoglobin recovery in patients who did not achieve CR. **a** Percentage of patients with durable (≥ 2 consecutive visits) recovery at any time on study. **b** Percentage of patients with hemoglobin recovery after the first, second, and third treatment cycles. For treatment cycle analysis, one threshold measurement was required; all patients were included regardless of their BL levels but each cycle only included remaining patients at risk in that cycle. Analysis set, *N* = number of patients with hemoglobin results in the cycle; patients, *n* = number of patients meeting recovery criteria in the cycle. Abbreviations: BL, baseline; CR, complete remission; LDAC, low-dose cytarabine

**Fig. 4:**
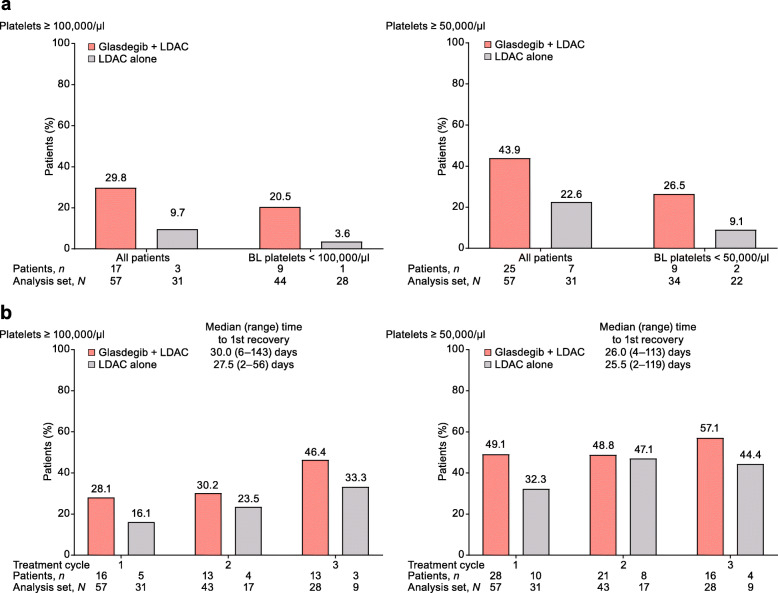
Platelet recovery in patients who did not achieve CR. **a** Percentage of patients with durable (≥ 2 consecutive visits) recovery at any time on study. **b** Percentage of patients with platelet recovery after the first, second, and third treatment cycles. For treatment cycle analysis, one threshold measurement was required; all patients were included regardless of their BL levels but each cycle only included remaining patients at risk in that cycle. Analysis set, *N* = number of patients with platelet results in the cycle; patients, *n* = number of patients meeting recovery criteria in the cycle. Abbreviations: BL, baseline; CR, complete remission; LDAC, low-dose cytarabine

**Fig. 5:**
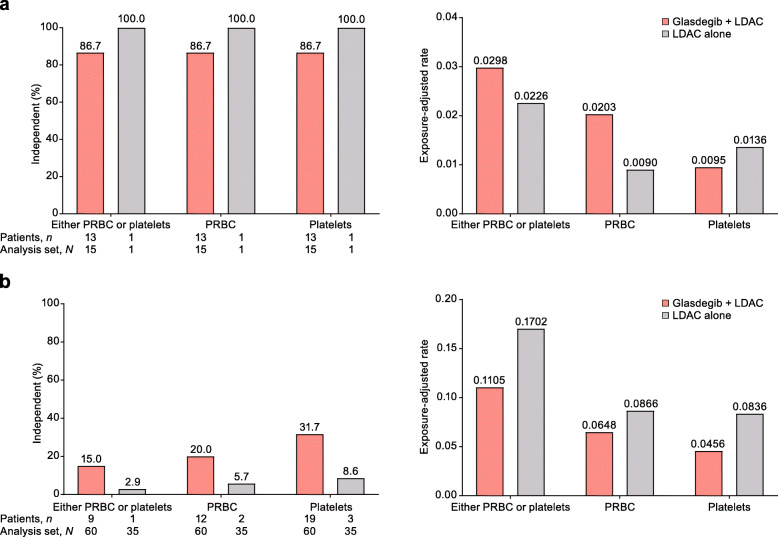
Transfusion independence and exposure-adjusted transfusion rates. **a** Patients who achieved CR. **b** Patients who did not achieve CR. Transfusion-independent patients are defined as those who have ≥ 8 weeks (56 days) without any type of transfusion at any point of the study. All other patients are considered transfusion-dependent. Analysis set, *N* = number of patients with platelet results in the cycle; patients, *n* = number of patients meeting recovery criteria in the cycle. Abbreviations: CR, complete remission; PRBC, packed red blood cells

Among patients who did not achieve CR, more patients achieved durable (≥ 2 consecutive visits) recovery of ANC, hemoglobin, and platelets in the glasdegib + LDAC arm than in the LDAC alone arm (part a of Figs. 2, 3, and 4). The median time to recovery with glasdegib + LDAC versus LDAC alone was longer for ANC (≥ 1000/μl, 21 vs 12 days; ≥ 500/μl, 14 vs 11 days), shorter for hemoglobin (≥ 10 g/dl, 25 vs 33 days; ≥ 9 g/dl, 12 vs 21 days), and similar for platelets (≥ 100,000/μl, 30 vs 28 days; ≥ 50,000/μl, 26 vs 26 days) (part b of Figs. 2, 3, and 4). Recovery was seen as early as cycle 1 in a meaningful proportion of patients, and counts were maintained or continued to improve during subsequent cycles in the remaining patients at risk (Additional file 4: Fig. S4). In addition, among patients who did not achieve CR, transfusion independence was achieved by 9/60 patients (15.0%) receiving glasdegib + LDAC and 1/35 patients (2.9%) receiving LDAC alone (Fig. 5).

### Safety

To account for imbalances in treatment duration between the glasdegib + LDAC and LDAC alone arms, AEs are presented separately for the first 90 days. All of the patients randomized and treated in both arms reported treatment-emergent AEs (TEAEs) during the course of the study. The incidence of TEAEs (any grade) was lower over the long term (after 90 days) than the short term (during the first 90 days) in patients receiving glasdegib + LDAC, regardless of whether they achieved CR or not (Table 6, Additional file 6: Table S2).

**Table 6:** All-causality treatment-emergent adverse events

	Achieved CR		Did not achieve CR	
*N* (%)	Glasdegib + LDAC	LDAC alone	Glasdegib + LDAC	LDAC alone
**During the first 90 days**	***n*= 15**	***n*= 1**	***n*= 60**	***n*= 35**
AEs	15 (100)	1 (100)	59 (98.3)	35 (100)
Serious AEs	10 (66.7)	0	39 (65.0)	26 (74.3)
Grade 3 or 4 AEs	14 (93.3)	0	49 (81.7)	33 (94.3)
Grade 5 AEs	0	0	12 (20.0)	13 (37.1)
Discontinued due to AEs	1 (6.7)	0	16 (26.7)	12 (34.3)
Glasdegib dose reduced due to AEs	2 (13.3)	N/A	6 (10.0)	N/A
Backbone chemotherapy dose reduced due to AEs	2 (13.3)	0	3 (5.0)	0
Glasdegib temporary discontinuation due to AEs	8 (53.3)	N/A	30 (50.0)	N/A
Backbone chemotherapy temporary discontinuation due to AEs	4 (26.7)	0	16 (26.7)	9 (25.7)
**After 90 days**	***n*= 14**	***n*= 1**	***n*= 29**	***n*= 13**
AEs	13 (92.9)	1 (100)	23 (79.3)	9 (69.2)
Serious AEs	6 (42.9)	0	16 (55.2)	7 (53.8)
Grade 3 or 4 AEs	10 (71.4)	1 (100)	20 (69.0)	7 (53.8)
Grade 5 AEs	1 (7.1)	0	9 (31.0)	3 (23.1)
Discontinued due to AEs	3 (21.4)	0	8 (27.6)	5 (38.5)
Glasdegib dose reduced due to AEs	4 (28.6)	N/A	1 (3.4)	N/A
Backbone chemotherapy dose reduced due to AEs	4 (28.6)	0	3 (10.3)	0
Glasdegib temporary discontinuation due to AEs	9 (64.3)	N/A	8 (27.6)	N/A
Backbone chemotherapy temporary discontinuation due to AEs	9 (64.3)	1 (100)	6 (20.7)	3 (23.1)

*AE* adverse event, *CR* complete remission, *LDAC* low-dose cytarabine, *N/A* not applicable

AEs thought to be linked to the inhibition of the Hedgehog signaling pathway in normal tissue appeared to occur less frequently in patients receiving glasdegib + LDAC who did not achieve CR versus those who achieved CR in the short term (alopecia, 1.7% vs 13.3%; dysgeusia, 13.3% vs 46.7%; muscle spasms, 8.3% vs 40.0%) and long term (alopecia, 6.9% vs 14.3%; dysgeusia, 6.9% vs 28.6%; muscle spasms, 10.3% vs 50.0%). However, the respective exposure-adjusted rates were similar both in the short term (alopecia, 0.0004 vs 0.0002; dysgeusia, 0.0015 vs 0.0017; muscle spasms, 0.0012 vs 0.0010) and long term (alopecia, 0.0001 vs 0.0001; dysgeusia, 0.0003 vs 0.0001; muscle spasms, 0.0005 vs 0.0002).

Among patients who did not achieve CR, the incidence of TEAEs associated with cytopenias, bleeding, and infections generally did not appear worse with glasdegib + LDAC versus LDAC alone either in the short term (anemia, 41.7% vs 42.9%; decreased neutrophil count, 5.0% vs 2.9%; decreased platelet count, 11.7% vs 11.4%; febrile neutropenia, 28.3% vs 22.9%; hemorrhage, 11.7% vs 28.6%; neutropenia, 6.7% vs 14.3%; pneumonia, 20.0% vs 25.7%; sepsis, 5.0% vs 14.3%; thrombocytopenia, 28.3% vs 25.7%) or the long term (anemia, 27.6% vs 23.1%; decreased neutrophil count, 3.4% vs 0%; decreased platelet count, 10.3% vs 7.7%; febrile neutropenia, 13.8% vs 7.7%; hemorrhage, 13.8% vs 0%; neutropenia, 20.7% vs 0%; pneumonia, 17.2% vs 23.1%; sepsis, 3.4% vs 7.7%; thrombocytopenia, 27.6% vs 15.4%). Decreased hemoglobin was not reported at any time during the study.

## Discussion

This post hoc analysis of the BRIGHT 1003 AML study extends previously reported results that show superior OS in patients with AML receiving glasdegib + LDAC versus LDAC alone, by demonstrating an improvement in survival in both patients who achieved CR and those who did not achieve CR. Furthermore, improvement was consistent across groups stratified by cytogenetic risk. The median OS among patients who achieved CR in the glasdegib + LDAC arm was > 2 years. This improvement in survival was despite the specified criteria to select patients who were ineligible for intensive chemotherapy in the study, resulting in a patient population with poor prognostic features (e.g., older age). Despite the significant improvement in response rate for patients with glasdegib + LDAC, the survival benefit appears disproportionate to the rate of CR, which was < 30%. Thus, we speculated that there could have been a benefit in survival even among patients who did not achieve CR. The present analysis suggests this was indeed the case.

The concept of CR being the only outcome associated with a survival benefit is applicable to intensive chemotherapy but not necessarily to other non-intensive treatment modalities. Although comparisons between trials should be considered with caution due to methodologic differences, the survival benefit of glasdegib + LDAC in patients who did not achieve CR is in line with previously reported outcomes for non-responders with other non-intensive AML therapies; median OS for non-responders (no CR) with enasidenib was 5.7 months and for non-responders (no CR or CRi) with venetoclax + LDAC, 3.5 months [8, 9]. A survival benefit in the absence of remission has been shown with agents such as azacitidine versus current commonly used treatments (median OS, 6.9 vs 4.2 months) [10].

A survival benefit in the absence of CR is accordant with the potential mechanism of action for glasdegib, which is believed to target cancer stem cells. Leukemic stem cells drive the initiation of AML and are typically resistant to conventional chemotherapy, which ultimately leads to relapse [11]. Mathematical modeling has demonstrated that stem cell proliferation and self-renewal may have a greater impact on disease progression than leukemic blasts, with high stem cell proliferation and self-renewal associated with low OS rates [12]. Preclinical studies have demonstrated that glasdegib directly inhibits leukemic stem cells and is synergistic with chemotherapeutic agents [4, 13]. An improvement in patient survival in the absence of remission has previously been reported with a number of stem cell-directed therapies (e.g., gemtuzumab ozogamicin) [11]. These studies further illustrate that response rate alone may not be an adequate marker of efficacy for stem cell-targeted therapies.

Another important finding in this analysis is the reduced risk of cytopenias in patients who did not achieve CR, with improved recovery of ANC, platelets, and hemoglobin with glasdegib + LDAC versus LDAC alone. Recovery of the three cell lineages was seen as early as cycle 1 in a meaningful proportion of patients. Additionally, more patients receiving glasdegib + LDAC were transfusion-independent. As the Hedgehog signaling pathway is not essential for normal adult hematopoiesis, this study demonstrates that treatment with glasdegib may target leukemic cells while limiting cytopenias and cytopenic complications [3, 4]. This is in contrast to many therapies for the treatment of patients with AML, which induce myelosuppression by affecting both normal and cancer cells. In addition to patients receiving glasdegib + LDAC requiring fewer transfusions, both glasdegib and LDAC can be administered at home, thereby reducing the need for hospitalization and visits to outpatient clinics in this patient population.

This study suggests that treatment with glasdegib + LDAC was associated with an acceptable safety profile in both patients who achieved CR and those who did not achieve CR. Importantly, among patients who did not achieve CR, little additional toxicity was seen with the addition of glasdegib to LDAC versus LDAC alone, and therefore, the survival and clinical benefits associated with glasdegib treatment occur without significant additional toxicity.

As expected in this older patient population which traditionally has median OS rates of 3–4 months, with long-term follow-up of BRIGHT 1003 AML (> 40 months), the majority of patients had discontinued treatment [14]. Overall, patients in both treatment arms predominately discontinued treatment due to insufficient response (glasdegib + LDAC, 43%; LDAC alone, 33%), with fewer patients discontinuing treatment due to AEs in the glasdegib + LDAC (17%) vs LDAC alone (28%) arm (Heuser M, et al: Clinical benefit of glasdegib plus low-dose cytarabine in patients with de novo and secondary acute myeloid leukemia: long-term analysis of a phase II randomized trial, submitted). The study protocol indicated patients should continue treatment as long as clinical benefit was observed. The best overall response with glasdegib + LDAC was frequently achieved late (after 5 cycles of therapy) into treatment (Fig S1), which lead to the recommendation that patients with AML are treated for a minimum of six cycles to allow time for a clinical response in the US Food and Drug Administration label [2]. Together, these data suggest that patients receiving glasdegib treatment should consider continuing treatment if seeing a clinical benefit, even in the absence of full hematologic remission.

The findings of this study may indicate that endpoints other than CR may be need to determine efficacy with stem cell-targeting agents, such as glasdegib, in this older patient population with AML as these may represent clinical benefit. Ongoing studies investigating the use of glasdegib in patients ineligible for intensive chemotherapy include endpoints such as overall survival, patient-reported outcomes to determine health-related quality of life, and transfusion independence [15–17].

## Conclusion

By targeting leukemic stem cells while sparing normal hematopoiesis, glasdegib + LDAC may be an effective agent for improving survival without substantial marrow suppression and attendant cytopenic complications. Together, these results may indicate that endpoints other than CR may be needed to determine efficacy with stem cell–targeting agents, with potential survival and clinical benefits seen in the absence of CR. Patients receiving glasdegib + LDAC seeing a clinical benefit should consider continuing treatment, even in the absence of cytologic remission. Glasdegib is being studied in a phase 3 clinical study for the treatment of AML (ClinicalTrials.gov, NCT03416179) in combination with azacitidine or 7 + 3 intensive chemotherapy [15].

## Supplementary information

**Additional file 1: Fig. S1.** Duration of treatment with best overall response. **a** For patients receiving glasdegib + LDAC. **b** For patients receiving LDAC alone. Abbreviations: CR, complete remission; CRi, CR with incomplete hematologic response; EOT, end of treatment; LDAC, low-dose cytarabine; MR, minor response; PR, partial response; SD, stable disease

**Additional file 2: Fig. S2.** Kaplan–Meier plots of OS. **a** In patients who achieved CR or CRi. **b** In patients who did not achieve CR or CRi. Abbreviations: CI, confidence interval; CR, complete remission; CRi, CR with incomplete hematologic response; LDAC, low-dose cytarabine; OS, overall survival

**Additional file 3: Fig. S3.** Kaplan–Meier plots of OS with censoring for systemic follow-up therapies. **a** In patients who achieved CR. **b** In patients who did not achieve CR. Abbreviations: CI, confidence interval; CR, complete remission; LDAC, low-dose cytarabine; N/E, not evaluable; OS, overall survival

**Additional file 4: Fig S4.** Blood count recovery in patients who did not achieve CR. Percentage of patients with **a.** ANC **b**. hemoglobin **c**. platelets recovery during cycles one to ten. One threshold measurement was required; all patients were included regardless of their BL levels but each cycle only included remaining patients at risk in that cycle. Analysis set, *N* = number of patients with ANC results in the cycle; patients, *n* = number of patients meeting recovery criteria in the cycle. Abbreviations: ANC, absolute neutrophil count; BL, baseline; CR, complete remission; LDAC, low-dose cytarabine.

**Additional file 5: Table S1.** Patient disposition

**Additional file 6: Table S2.** Treatment-emergent all-causality adverse events* occurring during first 90 days and after 90 days of therapy

## Data Availability

Upon request, and subject to certain criteria, conditions**,** and exceptions (see https**://**www.pfizer.com/science/clinical-trials/trial-data-and-results for more information), Pfizer will provide access to individual de-identified participant data from Pfizer-sponsored global interventional clinical studies conducted for medicines, vaccines**,** and medical devices (1) for indications that have been approved in the USA and/or EU or (2) in programs that have been terminated (i.e., development for all indications has been discontinued). Pfizer will also consider requests for the protocol, data dictionary, and statistical analysis plan. Data may be requested from Pfizer trials 24 months after study completion. The de-identified participant data will be made available to researchers whose proposals meet the research criteria and other conditions, and for which an exception does not apply, via a secure portal. To gain access, data requestors must enter into a data access agreement with Pfizer.

## Abbreviations

AE: Adverse event
AML: Acute myeloid leukemia
ANC: Absolute neutrophil count
BL: Baseline
CI: Confidence interval
CR: Complete remission
CRi: Complete remission with incomplete hematologic recovery
ECOG PS: Eastern Cooperative Oncology Group performance status
ELN: European LeukemiaNet
EOT: End of treatment
HR: Hazard ratio
LDAC: Low-dose cytarabine
LSC: Leukemic stem cell
MLFS: Morphologic leukemia-free state
MR: Minor response
N/A: Not applicable
N/E: Not evaluable
OS: Overall survival
PR: Partial remission
PRBC: Packed red blood cells
PRi: Partial remission with incomplete hematologic recovery
SD: Stable disease
SMO: Smoothened
TEAE: Treatment-emergent adverse event
