# Supplementary material for: Survival outcomes and clinical benefit in patients with acute myeloid leukemia treated with glasdegib and low-dose cytarabine according to response to therapy
Source: J Hematol Oncol. 2020 Jul 14;13:92. doi: 10.1186/s13045-020-00929-8 (PMC7362563; doi:10.1186/s13045-020-00929-8)
Supplement: Supplementary file 1 — Additional file 1: Fig. S1. Duration of treatment with best overall response. a For patients receiving glasdegib + LDAC. b For patients receiving LDAC alone. Abbreviations: CR, complete remission; CRi, CR with incomplete hematologic response; EOT, end of treatment; LDAC, low-dose cytarabine; MR, minor response; PR, partial response; SD, stable disease [file 13045_2020_929_MOESM1_ESM.pdf]

Fig. S1. Duration of treatment with best overall response. **a** For patients receiving glasdegib + LDAC. **b** For patients receiving LDAC alone. Abbreviations: CR, complete remission; CRi, CR with incomplete hematologic response; EOT, end of treatment; LDAC, low-dose cytarabine; MR, minor response; PR, partial response; SD, stable disease

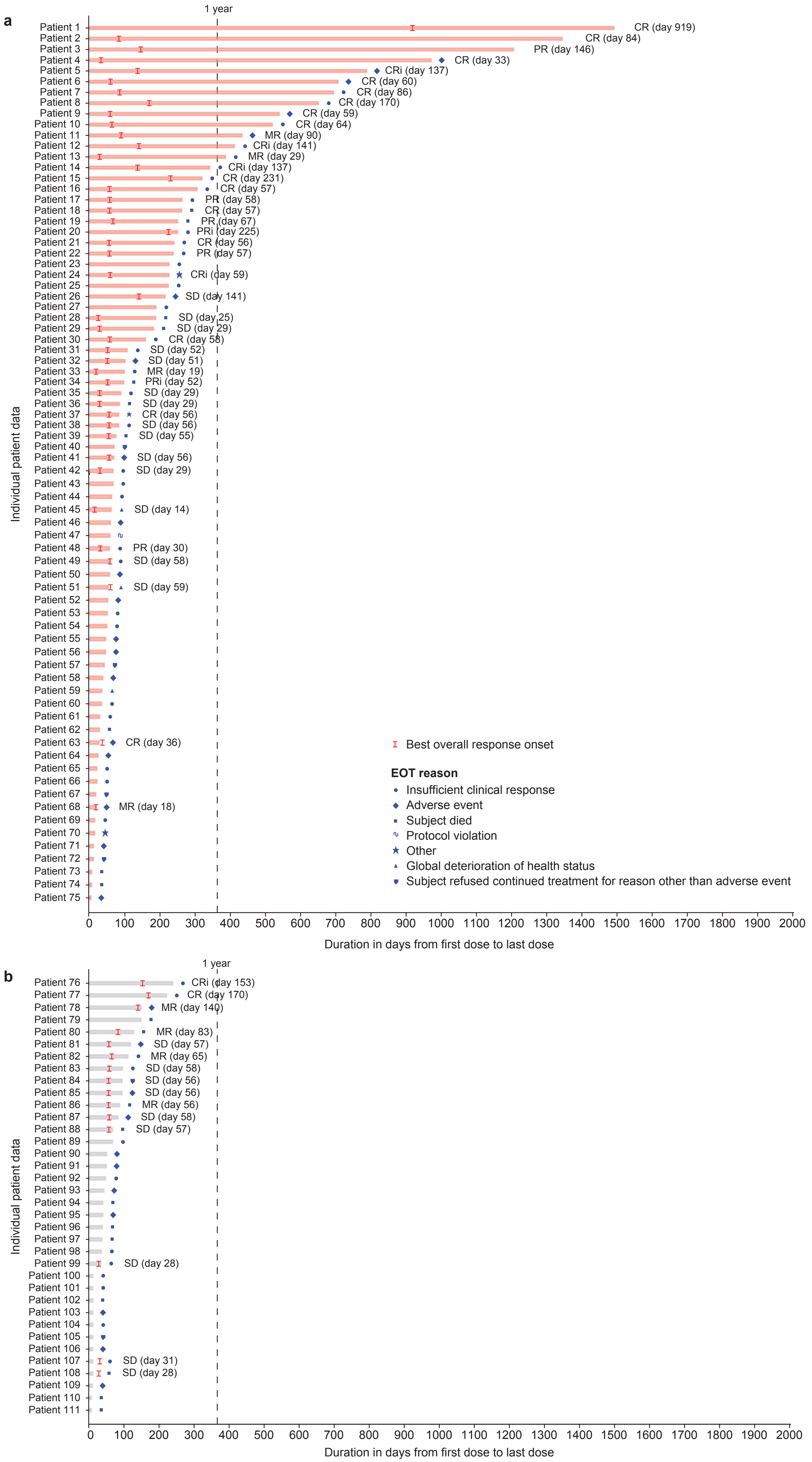
a
1 year
Individual patient data
Patient 1 CR (day 919)
Patient 2 CR (day 84)
Patient 3 PR (day 146)
Patient 4 CR (day 33)
Patient 5 CRi (day 137)
Patient 6 CR (day 60)
Patient 7 CR (day 86)
Patient 8 CR (day 170)
Patient 9 CR (day 59)
Patient 10 CR (day 64)
Patient 11 MR (day 90)
Patient 12 CRi (day 141)
Patient 13 MR (day 29)
Patient 14 CRi (day 137)
Patient 15 CR (day 231)
Patient 16 CR (day 57)
Patient 17 PR (day 58)
Patient 18 CR (day 57)
Patient 19 PR (day 67)
Patient 20 PRi (day 225)
Patient 21 CR (day 56)
Patient 22 PR (day 57)
Patient 23
Patient 24 CRi (day 59)
Patient 25
Patient 26 SD (day 141)
Patient 27
Patient 28 SD (day 25)
Patient 29 SD (day 29)
Patient 30 CR (day 58)
Patient 31 SD (day 52)
Patient 32 SD (day 51)
Patient 33 MR (day 19)
Patient 34 PRi (day 52)
Patient 35 SD (day 29)
Patient 36 SD (day 29)
Patient 37 CR (day 56)
Patient 38 SD (day 56)
Patient 39 SD (day 55)
Patient 40
Patient 41 SD (day 56)
Patient 42 SD (day 29)
Patient 43
Patient 44
Patient 45 SD (day 14)
Patient 46
Patient 47
Patient 48 PR (day 30)
Patient 49 SD (day 58)
Patient 50
Patient 51 SD (day 59)
Patient 52
Patient 53
Patient 54
Patient 55
Patient 56
Patient 57
Patient 58
Patient 59
Patient 60
Patient 61
Patient 62
Patient 63 CR (day 36)
Patient 64
Patient 65
Patient 66
Patient 67
Patient 68 MR (day 18)
Patient 69
Patient 70
Patient 71
Patient 72
Patient 73
Patient 74
Patient 75
Best overall response onset
EOT reason
Insufficient clinical response
Adverse event
Subject died
Protocol violation
Other
Global deterioration of health status
Subject refused continued treatment for reason other than adverse event
0 100 200 300 400 500 600 700 800 900 1000 1100 1200 1300 1400 1500 1600 1700 1800 1900 2000
Duration in days from first dose to last dose
b
1 year
Individual patient data
Patient 76 CRi (day 153)
Patient 77 CR (day 170)
Patient 78 MR (day 140)
Patient 79
Patient 80 MR (day 83)
Patient 81 SD (day 57)
Patient 82 MR (day 65)
Patient 83 SD (day 58)
Patient 84 SD (day 56)
Patient 85 SD (day 56)
Patient 86 MR (day 56)
Patient 87 SD (day 58)
Patient 88 SD (day 57)
Patient 89
Patient 90
Patient 91
Patient 92
Patient 93
Patient 94
Patient 95
Patient 96
Patient 97
Patient 98
Patient 99 SD (day 28)
Patient 100
Patient 101
Patient 102
Patient 103
Patient 104
Patient 105
Patient 106
Patient 107 SD (day 31)
Patient 108 SD (day 28)
Patient 109
Patient 110
Patient 111
0 100 200 300 400 500 600 700 800 900 1000 1100 1200 1300 1400 1500 1600 1700 1800 1900 2000
Duration in days from first dose to last dose
