# Supplementary material for: Survival outcomes and clinical benefit in patients with acute myeloid leukemia treated with glasdegib and low-dose cytarabine according to response to therapy
Source: J Hematol Oncol. 2020 Jul 14;13:92. doi: 10.1186/s13045-020-00929-8 (PMC7362563; doi:10.1186/s13045-020-00929-8)
Supplement: Supplementary file 2 — Additional file 2: Fig. S2. Kaplan–Meier plots of OS. a In patients who achieved CR or CRi. b In patients who did not achieve CR or CRi. Abbreviations: CI, confidence interval; CR, complete remission; CRi, CR with incomplete hematologic response; LDAC, low-dose cytarabine; OS, overall survival [file 13045_2020_929_MOESM2_ESM.pdf]

Fig. S2. Kaplan–Meier plots of OS. **a** In patients who achieved CR or CRi. **b** In patients who did not achieve CR or CRi. Abbreviations: CI, confidence interval; CR, complete remission; CRi, CR with incomplete hematologic response; LDAC, low-dose cytarabine; OS, overall survival

a

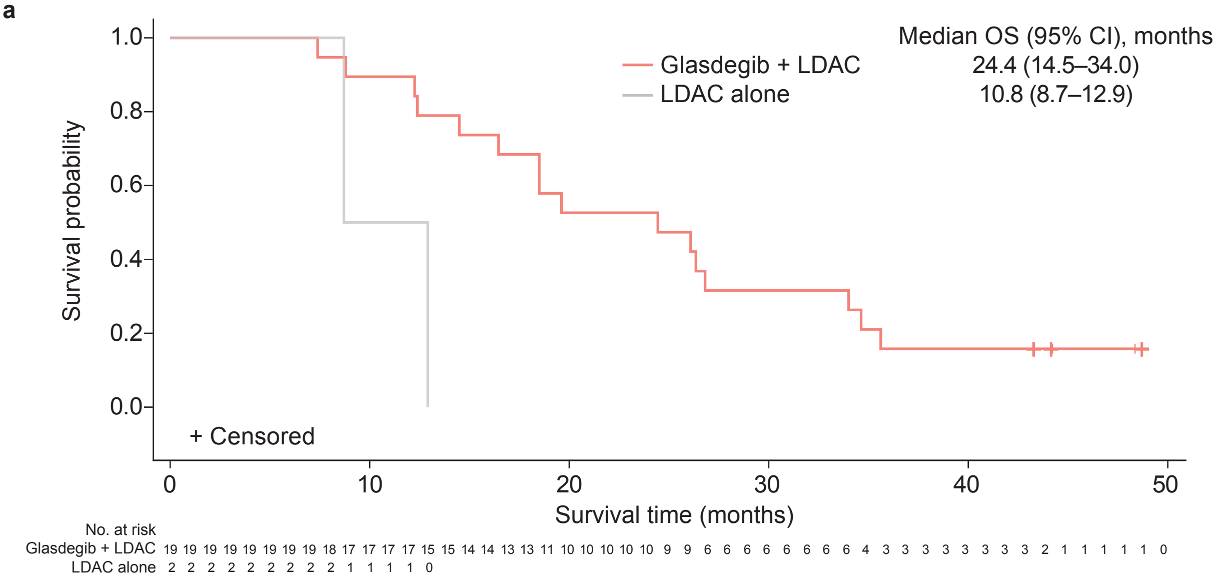
Median OS (95% CI), months
Glasdegib + LDAC 24.4 (14.5–34.0)
LDAC alone 10.8 (8.7–12.9)
Survival probability
1.0
0.8
0.6
0.4
0.2
0.0
+ Censored
0 10 20 30 40 50
Survival time (months)
No. at risk
Glasdegib + LDAC 19 19 19 19 19 19 19 19 18 17 17 17 17 15 15 14 14 13 13 11 10 10 10 10 10 9 9 6 6 6 6 6 6 6 4 3 3 3 3 3 3 3 3 2 1 1 1 1 1 0
LDAC alone 2 2 2 2 2 2 2 2 2 1 1 1 1 0

b

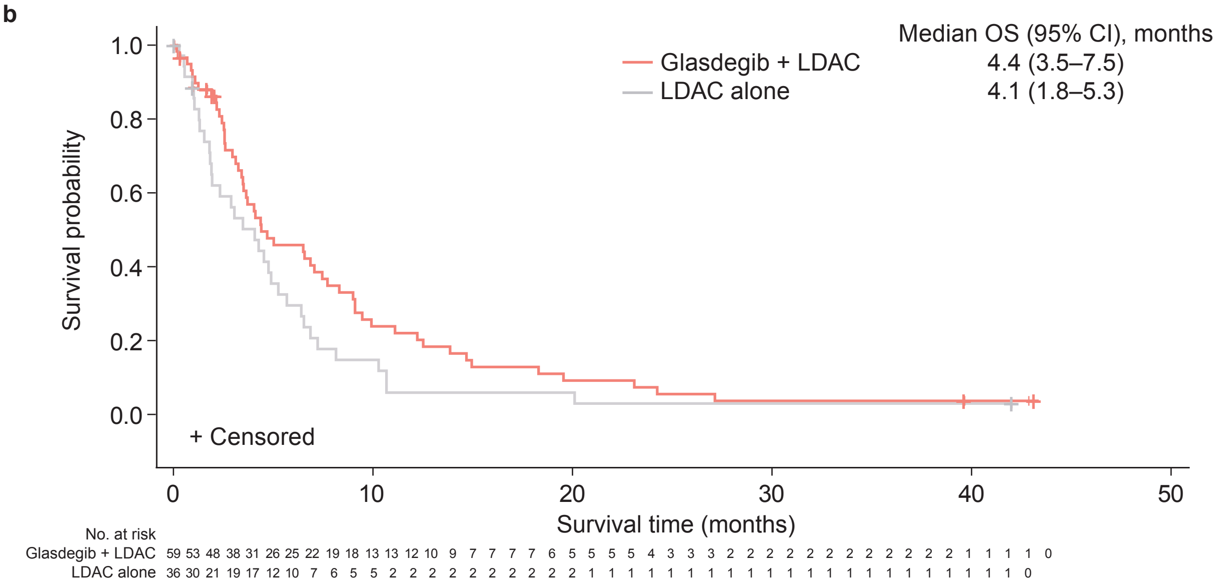
Median OS (95% CI), months
Glasdegib + LDAC 4.4 (3.5–7.5)
LDAC alone 4.1 (1.8–5.3)
Survival probability
1.0
0.8
0.6
0.4
0.2
0.0
+ Censored
0 10 20 30 40 50
Survival time (months)
No. at risk
Glasdegib + LDAC 59 53 48 38 31 26 25 22 19 18 13 13 12 10 9 7 7 7 7 6 5 5 5 5 4 3 3 3 2 2 2 2 2 2 2 2 2 2 2 2 1 1 1 1 0
LDAC alone 36 30 21 19 17 12 10 7 6 5 5 2 2 2 2 2 2 2 2 2 2 1 1 1 1 1 1 1 1 1 1 1 1 1 1 1 1 1 1 1 1 1 1 0
