# Supplementary material for: Survival outcomes and clinical benefit in patients with acute myeloid leukemia treated with glasdegib and low-dose cytarabine according to response to therapy
Source: J Hematol Oncol. 2020 Jul 14;13:92. doi: 10.1186/s13045-020-00929-8 (PMC7362563; doi:10.1186/s13045-020-00929-8)
Supplement: Supplementary file 3 — Additional file 3: Fig. S3. Kaplan–Meier plots of OS with censoring for systemic follow-up therapies. a In patients who achieved CR. b In patients who did not achieve CR. Abbreviations: CI, confidence interval; CR, complete remission; LDAC, low-dose cytarabine; N/E, not evaluable; OS, overall survival [file 13045_2020_929_MOESM3_ESM.pdf]

Fig. S3. Kaplan–Meier plots of OS with censoring for systemic follow-up therapies. **a** In patients who achieved CR. **b** In patients who did not achieve CR. Abbreviations: CI, confidence interval; CR, complete remission; LDAC, low-dose cytarabine; N/E, not evaluable; OS, overall survival

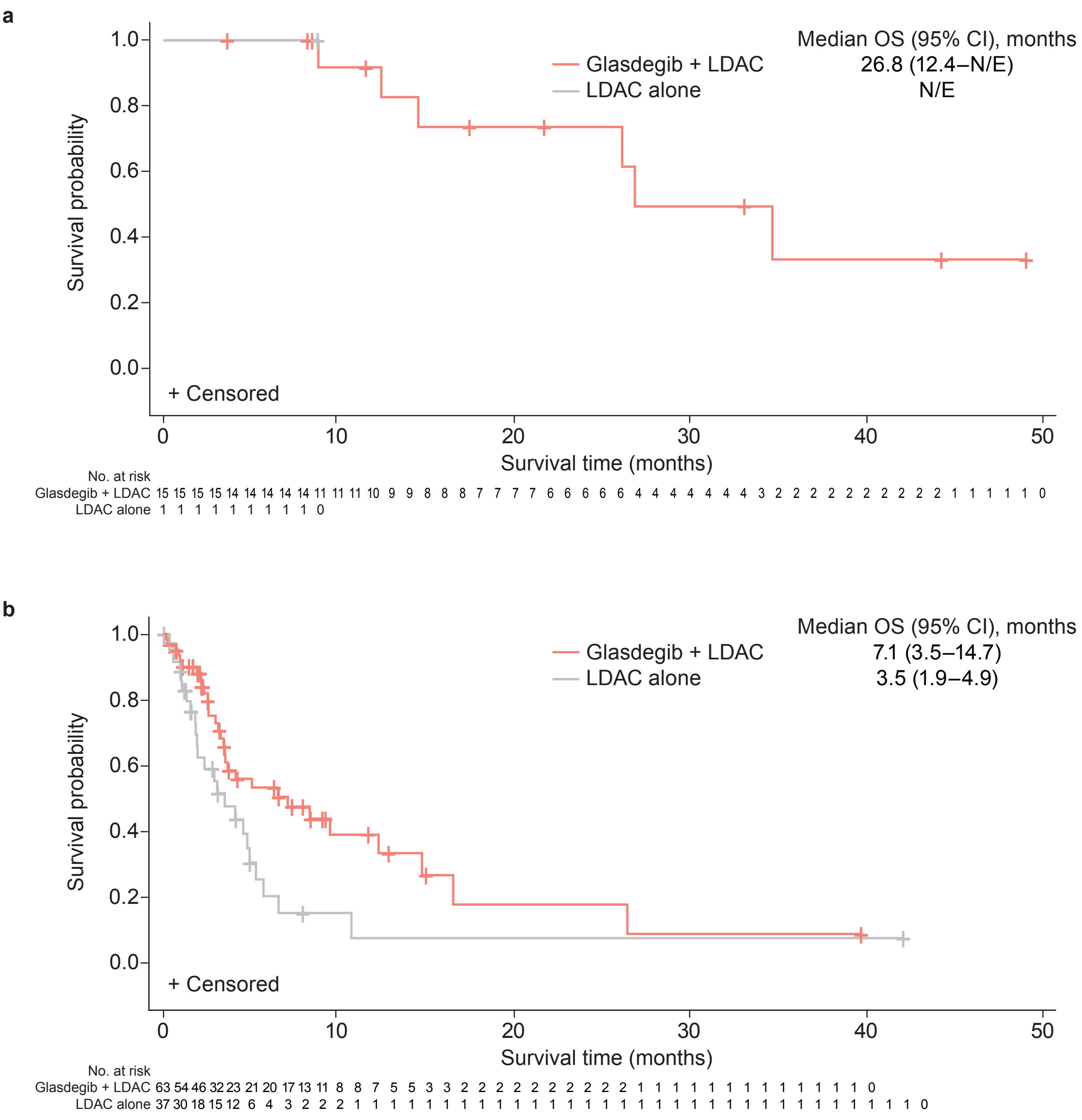
a
Median OS (95% CI), months
Glasdegib + LDAC 26.8 (12.4–N/E)
LDAC alone N/E
Survival probability
1.0
0.8
0.6
0.4
0.2
0.0
+ Censored
0
10
20
30
40
50
Survival time (months)
No. at risk
Glasdegib + LDAC 15 15 15 15 14 14 14 14 14 11 11 11 10 9 9 8 8 8 7 7 7 7 6 6 6 6 6 4 4 4 4 4 4 4 3 2 2 2 2 2 2 2 2 2 2 1 1 1 1 1 0
LDAC alone 1 1 1 1 1 1 1 1 1 0
b
Median OS (95% CI), months
Glasdegib + LDAC 7.1 (3.5–14.7)
LDAC alone 3.5 (1.9–4.9)
Survival probability
1.0
0.8
0.6
0.4
0.2
0.0
+ Censored
0
10
20
30
40
50
Survival time (months)
No. at risk
Glasdegib + LDAC 63 54 46 32 23 21 20 17 13 11 8 8 7 5 5 3 3 2 2 2 2 2 2 2 2 2 2 1 1 1 1 1 1 1 1 1 1 1 1 1 0
LDAC alone 37 30 18 15 12 6 4 3 2 2 2 1 1 1 1 1 1 1 1 1 1 1 1 1 1 1 1 1 1 1 1 1 1 1 1 1 1 1 1 1 1 1 1 0
