# Supplementary material for: Survival outcomes and clinical benefit in patients with acute myeloid leukemia treated with glasdegib and low-dose cytarabine according to response to therapy
Source: J Hematol Oncol. 2020 Jul 14;13:92. doi: 10.1186/s13045-020-00929-8 (PMC7362563; doi:10.1186/s13045-020-00929-8)
Supplement: Supplementary file 4 — Additional file 4: Fig S4. Blood count recovery in patients who did not achieve CR. Percentage of patients with a. ANC b. hemoglobin c. platelets recovery during cycles one to ten. One threshold measurement was required; all patients were included regardless of their BL levels but each cycle only included remaining patients at risk in that cycle. Analysis set, N = number of patients with ANC results in the cycle; patients, n = number of patients meeting recovery criteria in the cycle. Abbreviations: ANC, absolute neutrophil count; BL, baseline; CR, complete remission; LDAC, low-dose cytarabine. [file 13045_2020_929_MOESM4_ESM.pdf]

Fig S4. Blood count recovery in patients who did not achieve CR. Percentage of patients with **a.** ANC **b**. hemoglobin **c**. platelets recovery during cycles one to ten. One threshold measurement was required; all patients were included regardless of their BL levels but each cycle only included remaining patients at risk in that cycle. Analysis set, N = number of patients with ANC results in the cycle; patients, n = number of patients meeting recovery criteria in the cycle. Abbreviations: ANC, absolute neutrophil count; BL, baseline; CR, complete remission; LDAC, low-dose cytarabine.

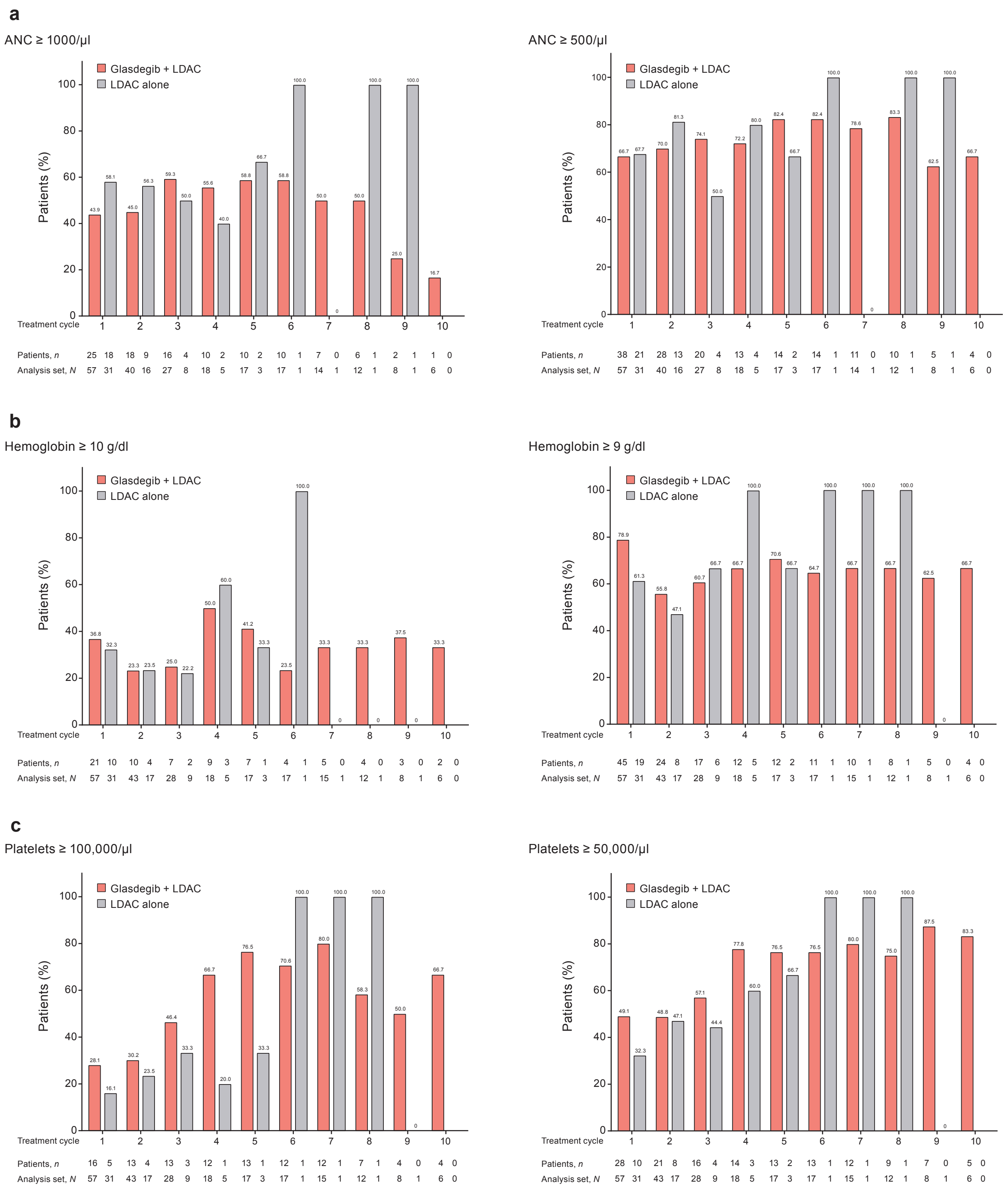
a
ANC ≥ 1000/µl
Glasdegib + LDAC
LDAC alone
Patients (%)
Treatment cycle
43.9 58.1 45.0 56.3 59.3 50.0 55.6 40.0 58.8 66.7 58.8 100.0 50.0 0 50.0 100.0 25.0 100.0 16.7
Patients, n 25 18 18 9 16 4 10 2 10 2 10 1 7 0 6 1 2 1 1 0
Analysis set, N 57 31 40 16 27 8 18 5 17 3 17 1 14 1 12 1 8 1 6 0
ANC ≥ 500/µl
66.7 67.7 70.0 81.3 74.1 50.0 72.2 80.0 82.4 66.7 82.4 100.0 78.6 0 83.3 100.0 62.5 100.0 66.7
Patients, n 38 21 28 13 20 4 13 4 14 2 14 1 11 0 10 1 5 1 4 0
Analysis set, N 57 31 40 16 27 8 18 5 17 3 17 1 14 1 12 1 8 1 6 0
b
Hemoglobin ≥ 10 g/dl
36.8 32.3 23.3 23.5 25.0 22.2 50.0 60.0 41.2 33.3 23.5 100.0 33.3 0 33.3 0 37.5 0 33.3
Patients, n 21 10 10 4 7 2 9 3 7 1 4 1 5 0 4 0 3 0 2 0
Analysis set, N 57 31 43 17 28 9 18 5 17 3 17 1 15 1 12 1 8 1 6 0
Hemoglobin ≥ 9 g/dl
78.9 61.3 55.8 47.1 60.7 66.7 66.7 100.0 70.6 66.7 64.7 100.0 66.7 100.0 66.7 100.0 62.5 0 66.7
Patients, n 45 19 24 8 17 6 12 5 12 2 11 1 10 1 8 1 5 0 4 0
Analysis set, N 57 31 43 17 28 9 18 5 17 3 17 1 15 1 12 1 8 1 6 0
c
Platelets ≥ 100,000/µl
28.1 16.1 30.2 23.5 46.4 33.3 66.7 20.0 76.5 33.3 70.6 100.0 80.0 100.0 58.3 100.0 50.0 0 66.7
Patients, n 16 5 13 4 13 3 12 1 13 1 12 1 12 1 7 1 4 0 4 0
Analysis set, N 57 31 43 17 28 9 18 5 17 3 17 1 15 1 12 1 8 1 6 0
Platelets ≥ 50,000/µl
49.1 32.3 48.8 47.1 57.1 44.4 77.8 60.0 76.5 66.7 76.5 100.0 80.0 100.0 75.0 100.0 87.5 0 83.3
Patients, n 28 10 21 8 16 4 14 3 13 2 13 1 12 1 9 1 7 0 5 0
Analysis set, N 57 31 43 17 28 9 18 5 17 3 17 1 15 1 12 1 8 1 6 0
