# Supplementary material for: Survival outcomes and clinical benefit in patients with acute myeloid leukemia treated with glasdegib and low-dose cytarabine according to response to therapy
Source: J Hematol Oncol. 2020 Jul 14;13:92. doi: 10.1186/s13045-020-00929-8 (PMC7362563; doi:10.1186/s13045-020-00929-8)
Supplement: Supplementary file 5 — Additional file 5: Table S1. Patient disposition [file 13045_2020_929_MOESM5_ESM.pdf]

Table S1. Patient disposition

	Achieved CR<br>*n* = 16		Did not achieve CR<br>*n* = 100	
	Glasdegib + LDAC	LDAC alone	Glasdegib + LDAC	LDAC alone
Assigned to study treatment, *n*	15	1	63	37
Treated, *n*	15	1	60	35
Treatment discontinued, *n* (%)	13 (86.7)	1 (100)	59 (98.3)	35 (100)
Treatment ongoing at date of data cut-off, *n* (%)	2 (13.3)	0	1 (1.7)	0
Study discontinued, *n* (%)	12 (80.0)	1 (100)	61 (96.8)	36 (97.3)
Study ongoing at date of data cut-off, *n* (%)	3 (20.0)	0	2 (3.2)	1 (2.7)

CR, complete remission; LDAC, low-dose cytarabine
