# Supplementary material for: Survival outcomes and clinical benefit in patients with acute myeloid leukemia treated with glasdegib and low-dose cytarabine according to response to therapy
Source: J Hematol Oncol. 2020 Jul 14;13:92. doi: 10.1186/s13045-020-00929-8 (PMC7362563; doi:10.1186/s13045-020-00929-8)
Supplement: Supplementary file 6 — Additional file 6: Table S2. Treatment-emergent all-causality adverse events* occurring during first 90 days and after 90 days of therapy [file 13045_2020_929_MOESM6_ESM.pdf]

Table S2. Treatment-emergent all-causality adverse events* occurring during first 90 days and after 90 days of therapy

	Achieved CR				Did not achieve CR			
	Glasdegib + LDAC		LDAC alone		Glasdegib + LDAC		LDAC alone	
MedDRA preferred term, *n* (%)	*n* = 15		*n* = 1		*n* = 60		*n* = 35	
During the first 90 days	Any grade	Grade 3/4	Any grade	Grade 3/4	Any grade	Grade 3/4	Any grade	Grade 3/4
Anemia	8 (53.3)	7 (46.7)	0	0	25 (41.7)	24 (40.0)	15 (42.9)	13 (37.1)
Nausea	5 (33.3)	0	0	0	17 (28.3)	1 (1.7)	4 (11.4)	1 (2.9)
Febrile neutropenia	6 (40.0)	6 (40.0)	0	0	17 (28.3)	17 (28.3)	8 (22.9)	8 (22.9)
Decreased appetite	3 (20.0)	0	0	0	12 (20.0)	0	3 (8.6)	1 (2.9)
Thrombocytopenia	6 (40.0)	6 (40.0)	0	0	17 (28.3)	17 (28.3)	9 (25.7)	8 (22.9)
Diarrhea	6 (40.0)	0	0	0	7 (11.7)	1 (1.7)	9 (25.7)	0
Fatigue	5 (33.3)	1 (6.7)	0	0	14 (23.3)	6 (10.0)	6 (17.1)	2 (5.7)
Pneumonia	2 (13.3)	1 (6.7)	0	0	12 (20.0)	7 (11.7)	9 (25.7)	7 (20.0)
Pyrexia	3 (20.0)	1 (6.7)	0	0	12 (20.0)	0	8 (22.9)	1 (2.9)
Constipation	3 (20.0)	0	1 (100)	0	12 (20.0)	1 (1.7)	4 (11.4)	0

Edema peripheral	5 (33.3)	0	0	0	12 (20.0)	0	7 (20.0)	1 (2.9)
Dysgeusia	7 (46.7)	0	0	0	8 (13.3)	0	1 (2.9)	0
Vomiting	4 (26.7)	0	0	0	11 (18.3)	2 (3.3)	3 (8.6)	1 (2.9)
Cough	2 (13.3)	0	0	0	10 (16.7)	0	5 (14.3)	1 (2.9)
Dizziness	4 (26.7)	0	0	0	9 (15.0)	1 (1.7)	3 (8.6)	0
Dyspnea	1 (6.7)	0	0	0	12 (20.0)	4 (6.7)	9 (25.7)	2 (5.7)
Muscle spasm	6 (40.0)	0	0	0	5 (8.3)	0	2 (5.7)	0
Weight decreased	4 (26.7)	0	0	0	7 (11.7)	0	1 (2.9)	0

	Glasdegib + LDAC *n* = 14		LDAC alone *n* = 1		Glasdegib + LDAC *n* = 29		LDAC alone *n* = 13	
After 90 days	Any grade	Grade 3/4	Any grade	Grade 3/4	Any grade	Grade 3/4	Any grade	Grade 3/4
Anemia	5 (35.7)	3 (21.4)	0	0	8 (27.6)	7 (24.1)	3 (23.1)	3 (23.1)
Nausea	4 (28.6)	0	0	0	5 (17.2)	1 (3.4)	0	0
Febrile neutropenia	0	0	0	0	4 (13.8)	4 (13.8)	1 (7.7)	1 (7.7)
Decreased appetite	5 (35.7)	1 (7.1)	0	0	8 (27.6)	2 (6.9)	3 (23.1)	2 (15.4)
Thrombocytopenia	2 (14.3)	1 (7.1)	0	0	8 (27.6)	8 (27.6)	2 (15.4)	2 (15.4)

Diarrhea	9 (64.3)	1 (7.1)	0	0	5 (17.2)	2 (6.9)	1 (7.7)	1 (7.7)
Fatigue	5 (35.7)	2 (14.3)	0	0	2 (6.9)	1 (3.4)	2 (15.4)	0
Pneumonia	3 (21.4)	1 (7.1)	0	0	5 (17.2)	2 (6.9)	3 (23.1)	1 (7.7)
Pyrexia	5 (35.7)	1 (7.1)	0	0	5 (17.2)	0	1 (7.7)	1 (7.7)
Constipation	1 (7.1)	0	0	0	4 (13.8)	0	1 (7.7)	0
Edema peripheral	4 (28.6)	0	0	0	1 (3.4)	1 (3.4)	0	0
Dysgeusia	4 (28.6)	0	0	0	2 (6.9)	0	0	0
Vomiting	2 (14.3)	0	0	0	2 (6.9)	0	0	0
Cough	3 (21.4)	0	0	0	2 (6.9)	0	1 (7.7)	0
Dizziness	3 (21.4)	0	0	0	3 (10.3)	0	0	0
Dyspnea	2 (14.3)	0	0	0	2 (6.9)	0	2 (15.4)	0
Muscle spasm	7 (50.0)	3 (21.4)	0	0	3 (10.3)	1 (3.4)	0	0
Weight decreased	5 (35.7)	2 (14.3)	0	0	1 (3.4)	0	0	0

CR, complete remission; LDAC, low-dose cytarabine; MedDRA, Medical Dictionary for Regulatory Activities; TEAE, treatment-emergent adverse event

*Reported for TEAEs occurring in ≥ 20% of patients (receiving glasdegib + LDAC or LDAC alone) in the BRIGHT AML 1003 study (glasdegib + LDAC: *n* = 75; LDAC alone: *n* = 36)
